# Poly(pyrrole-*co-o*-toluidine) wrapped CoFe_2_O_4_/R(GO–OXSWCNTs) ternary composite material for Ga^3+^ sensing ability

**DOI:** 10.1039/c9ra03593a

**Published:** 2019-10-16

**Authors:** Dina F. Katowah, Mahmoud A. Hussein, M. M. Alam, T. R. Sobahi, M. A. Gabal, Abdullah M. Asiri, Mohammed M. Rahman

**Affiliations:** Chemistry Department, Faculty of Science, King Abdulaziz University P. O. Box 80203 Jeddah 21589 Saudi Arabia maabdo@kau.edu.sa mahmali@aun.edu.eg mahussein74@yahoo.com mmrahman@kau.edu.sa; Polymer Chemistry Lab, Chemistry Department, Faculty of Science, Assiut University Assiut 71516 Egypt; Center of Excellence for Advanced Materials Research (CEAMR), King Abdulaziz University Jeddah 21589 Saudi Arabia; Department of Chemical Engineering and Polymer Science, Shahjalal University of Science and Technology Sylhet 3100 Bangladesh; Chemistry Department, Faculty of Science, Benha University Benha Egypt

## Abstract

In this study, we report a novel ternary conductive hybrid material with high stability, conductivity, and excellent electrochemical Ga^3+^ sensing ability. Ternary poly(pyrrole-*co-o*-toluidine)/CoFe_2_O_4_/reduced graphene oxide–oxidized single-wall carbon nanotube nanocomposites in the form of P(Py-*co*-OT)/CF/R(GO–OXSWCNTs) NCs have been synthesized through an *in situ* chemical polymerization method *via* a facile three-step approach. Single phase CoFe_2_O_4_ (CF) nanoparticles (NPs) were synthesized using an egg white method, while reduced graphene oxide–oxidized single-wall carbon nanotubes R(GO–OXSWCNTs) were prepared *via* co-reduction of graphene oxide along with oxidized SWCNTs flowed by coating CF and R(GO–OXSWCNTs) with a poly(pyrrole-*co-o*-toluidine) matrix P(Py-*co*-OT) copolymer. The results of X-ray diffraction spectroscopy (XRD), Fourier-transform infrared spectroscopy (FTIR) and Raman indicated that the P(Py-*co*-OT)/CF/R(GO–OXSWCNTs) NCs were effectively synthesized with strong interactions among the constituents. The thermal stability of P(Py-*co*-OT)/CF/R(GO–OXSWCNTs) NCs is considerably enhanced in the composite format. Scanning electron microscopy (SEM), transmission electron microscopy (TEM) and atomic force microscopy (AFM) demonstrated that CF and R(GO–OXSWCNTs) were well coated by P(Py-*co*-OT). The electrical conductivity study showed that P(Py-*co*-OT) and R(GO–OXSWCNTs) might significantly improve the conductivity and the electrochemical performance of the CF. A Ga^3+^ ion selective electrochemical sensor was fabricated by coating a glassy carbon electrode (GCE) with synthesized P(Py-*co*-OT)/CF/R(GO–OXSWCNTs) NCs by using 5% Nafion binder. The slope of the calibration curve was used to calculate the sensor's analytical parameters, such as sensitivity (13.0569 μA μM^−1^ cm^−2^), detection limit (96.27 ± 4.81 pM), quantification limit (43.523 pM), response time, reproducibility, large linear dynamic range, and linearity. The validation of the P(Py-*co*-OT)/CF/R(GO–OXSWCNTs) NCs/GCE sensor probe was investigated by a standard addition method (recovery) in the presence of various environmental samples and satisfying results were obtained.

## Abstract 2

A ternary P(Py-*co*-OT)/CF/R(GO–OXSWCNTs) nanocomposite has been fabricated as a novel conductive hybrid material with high stability and excellent electrochemical Ga^3+^ sensing ability.

## Graphical abstract

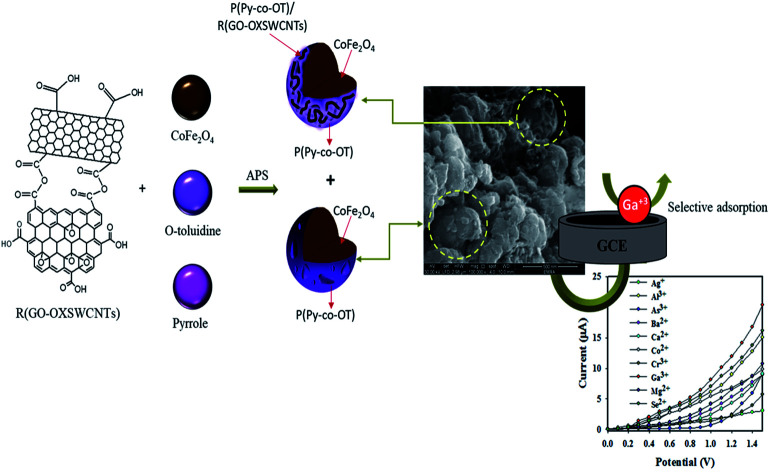

## 1. Introduction

Generally, the Ga^3+^ cation is a stable and non-polarizable Lewis base in aqueous solution and due to its excellent semi-conductive property, it has diverse industrial applications in the semi-conductor industry for making integrated electronic circuits. The extended pollution of the environment with gallium dust occurs in the semiconductor industrial areas. It is found as environmental contamination in the zinc and aluminum purification processes from their ores.^1,2^ The useful research on gallium to improve the fabrication of the semiconductor materials has increased the potential risk for toxicity to the environment and subsequently human health.^3,4^ Gallium with up-taken calcium is a strong inhibitor for the synthesis of protein, DNA and aminolevulinic acid dehydratase through an enzymatic pathway in humans.^5–7^ It has been reported that the gallium is responsible for depression of bone marrow, the toxicity of testicular and hemorrhagic nephritis.^8–10^ Besides this, the gallium has been found to accumulate in lymph nodes, in tumours, and cause inflammation of tissues and bone.^11,12^ Clinically, it is used as medicine to the treatment of various disease including hypercalcemia of malignancy^13^ osteoporosis^14^ Paget's disease of bone^15,16^ as well as bone pain associated with multiple myeloma and bone metastases.^17^ In the present diagnosis technology, the radioactive gallium isotopes (^67^Ga and ^68^Ga) are used to detect a tumor, particularly in the case of bone cancer.^1,2^ The body growth of teleost's and tilapia larvae also retardate in the presence of the gallium toxicity.^18^ The adverse toxic effect of gallium is not well distinct for animals living in aqueous. Therefore, a sensitive and quick detection method for Ga^3+^ cation is necessary. A number of analytical methods including electrochemical sensor,^19^ liquid chromatography,^20^ spectrophotometry^21^ and high-performance liquid chromatography (HPLC)^22^ are typically used a technique for confirming detection of Ga^3+^ ion. However, the electrochemical method based on electrochemical (*I*–*V*) approach using organometallic nanocomposites is the most convention and frequently implemented reliable and sensitive detection technique in short response time for heavy metal ions as described by previous authors.^23–26^ Conducting polymers CPs are important materials of significant interest in various applications such as corrosion inhibitors, organic electronics, batteries, sensors and electrochromic devices.^27^ CPs have useful applications in the development of chemical sensors.^28^ The great benefit of CPs in sensor applications is that CPs can be sensitive to small perturbations and might present improved response properties. Thus, CPs enhance the sensitivity of the sensors because of their electrical conductivity or charge transport features.^29^ Combining conductive fillers, such as carbon materials and a metal particle, into the matrix of the polymers can achieve electrical conductivity in the polymers.^30–34^ Conducting polymer nanocomposites (CPNs) are good candidates for different applications such as electronics, telecommunications,^35^ biosensors,^36,37^ fire retardants,^38^ electrocatalysts for fuel cells, electrodes for electrodeposition^39^ and the aerospace industry.^40^ The integration of magnetic particles into a polymer matrix has been a developing field during recent years, particularly when the material requires high conductivity. Materials with high conductivity as well as high magnetic permeability are required for various applications such as electronics, biosensors, electronic nonlinear optics, magnetic shielding, microwave absorbers and adsorption materials.^41–44^ Spinel ferrites are an important group of magnetic materials with a general formula of MFe_2_O_4_ (M = a divalent cation). Their properties, such as electrical, electronic, optical, catalytic and magnetic properties^45–49^ have gained attention in fundamental studies and technological applications.^50,51^ The conductivity of ferrite comes from the charge hopping of carriers within cations occupying the octahedral (oh) sites.^52^ Ferrites are preferably explored as sensing materials because they exhibit good chemical and thermal stabilities under operating conditions.^53^ Among the magnetic nanoparticles, cobalt ferrite exhibits a cubic spinel structure.^54^ The CF magnetic NPs have been extensively studied for the fabrication of biosensors due to its strong super-paramagnetic behaviour, biocompatibility and low toxicity.^55–57^ The combination of CPs and CF may lead to enhanced electrochemical performance. At present, carbon nanomaterials, for instance, carbon nanotubes (CNTs) and graphene (Gr), have become a topic of interest in sensors owing to their excellent electrical, mechanical and thermal properties along with their unique nanoscale structure. These properties are attractive for the improvement of the electrical conductivity of polymer-based composites.^58,59^ Currently, the many significant reviews on (Gr and/or CNTs) nanohybrid composites have identified many properties, such as excellent electrical and thermal conductivity, toughness and electrochemical performance, exhibited by these materials compared to those of neat (Gr or CNTs) composites.^60–63^ These composites have shown great potential for applications in various areas of nanotechnology research such as supercapacitors, batteries, biosensors, and optical devices.^64^ Integrating rGO and CNTs into a skeleton of conductive carbon can act as an electron transfer route into the matrix of the polymer.^65^ This hybrid structure offers advantageous properties that overcome some of the shortcomings of individual components. Based on earlier work, it is of great interest to study ternary P(Py-*co*-OT)/CF/R(GO–OXSWCNTs) NCs for the development of an electrochemical sensor. To the best of our knowledge, no studies on the synthesis of this interesting nanostructure have been reported so far. In the present paper, novel electromagnetic polymeric NCs were prepared by combining P(Py-*co*-OT), CF and R(GO–OXSWCNTs) NCs through an *in situ* chemical polymerization method. This composite can integrate the advantages of each component, leading to the improved electronic conductivity of NCs, thus resulting in the high performance of the sensor. The nanocomposites of ternary P(Py-*co*-OT)/CF/R(GO–OXSWCNTs) were used for the selective detection of Ga^3+^ cation in phosphate buffer. To fabricate the desired Ga^3+^ cationic sensor, the synthesized P(Py-*co*-OT)/CF/R(GO–OXSWCNTs) NCs were deposited on the flat part of a GCE with surface area of 0.0316 cm^2^. The analytical properties of the Ga^3+^ ion sensor were investigated. The proposed electrochemical sensor displayed outstanding sensor features such as sensitivity, LDR, DL, response time, reproducibility and stability. Finally, the sensing validity to analyse real environmental samples was performed and satisfactory results were obtained.

## 2. Experimental procedure

### 2.1. Materials

Pyrrole (Py), *o*-toluidine (OT), Co(NO_3_)_2_·6H_2_O and Fe(NO_3_)_3_·9H_2_O were obtained from Fluka, Switzerland. Graphene and SWCNTs were purchased from the XFNANO Advanced Materials Supplier Inc., China. Ammonium persulfate (APS) was purchased from Acros Organics. Hydrochloric acid (HCl) and the analytical chemicals, including Al_2_(SO_4_)_3_, CrCl_3_, Co(NO_3_)_2_, Ga(NO_3_)_3_, AsCl_3_, SeO, AgNO_3_, MgCl_2_, CaCl_2_ and Ba(NO_3_)_2_, were received from Sigma-Aldrich (USA) and used directly as received. Deionized water was used throughout. The auxillary chemicals, such as ammonium, monosodium and disodium phosphate buffer and Nafion (5% Nafion suspension in ethanol), were used to complete this study. Eggs were purchased from the local market.

### 2.2. Preparation of the CF

CF NPs were obtained through an egg white method according to the literature.^66^ Briefly, a homogeneous egg-white solution (60 mL of egg white (ovalbumin) with 40 mL of deionized water) and nitrate solution (a stoichiometric amount of Co(NO_3_)_2_·6H_2_O and Fe(NO_3_)_3_·9H_2_O with 40 mL of deionized water) were prepared at room temperature. A well-dissolved solution was obtained by added the nitrate solution to the egg-white solution under varying stirring at 100 °C. No pH adjustment was performed because the medium was alkaline. A discharged precursor was obtained by heating the mixed solution with continuous stirring for many hours. Finally, the obtained powder was calcined at 500 °C for 1 h.

### 2.3. Preparation of P(Py-*co*-OT)/CF NCs

P(Py-*co*-OT)/CF NCs were prepared by an *in situ* polymerization method.^67^ The typical preparation process is described as follows: 5% of the CF NPs were added into a three-neck flask (250 mL) containing 100 mL of 1 M HCl, followed by the addition of (0.67 mL, doubly distilled) of Py and (1.07 mL, doubly distilled) of OT. The mixture was ultrasonicated for 30 min. Then, the mixed solution was cooled to 0–4 °C in an ice bath with continuous stirring. A pre-cooled solution of (5 g) ammonium persulfate (APS) dissolved in 100 mL of 1 M HCl was then added dropwise into the above solution within approximately 30 min with constant stirring at 0–4 °C under a nitrogen atmosphere. The polymerization reaction was maintained at 0–4 °C in the nitrogen atmosphere for 24 h with constant stirring. The precipitate formed was collected by ultracentrifugation and washed with deionized water several times until the filtrate became colourless. Finally, the fine black powder was dried at 60 °C for 24 h.

### 2.4. Preparation of R(GO–OXSWCNTs) composite

The R(GO–OXSWCNTs) were chemically prepared by the co-reduction of oxidized SWCNTs and graphene oxide^66^ with a 1 : 1 mixing ratio as follows:

Graphene oxide was prepared according to a modified Hummers' method.^68^ An aqueous suspension of graphene oxide in deionized water was prepared by sonication of GO in an ultrasonic bath for 2 h. OXSWCNTs were prepared according to the literature.^69^ The SWCNTs (100 mg) were dispersed in a mixture of 70% nitric acid and 96% sulfuric acid (1 : 3 v/v) and ultrasonicated for 4 h. After sonication, the suspension was refluxed at 80 °C in an oil bath with magnetic stirring for 2 h. Then, the obtained mixture was diluted with deionized water and dialyzed in deionized water until the washing showed a pH > 5. The suspensions of GO and OXSWNTs were then mixed by ultrasonication for 2 h in a round-bottomed flask. Hydrazine monohydrate (50 μL for 150 mg of GO–OXSWCNTs) was added to the mixture, and the flask was immersed into an oil bath and refluxed with magnetic stirring at 98 °C for 24 h. The resulting black powder was filtered and rinsed with deionized water several times and dried under vacuum overnight.

### 2.5. Preparation of P(Py-*co*-OT)/CF/R(GO–OXSWCNTs) NCs

**Scheme 1:**
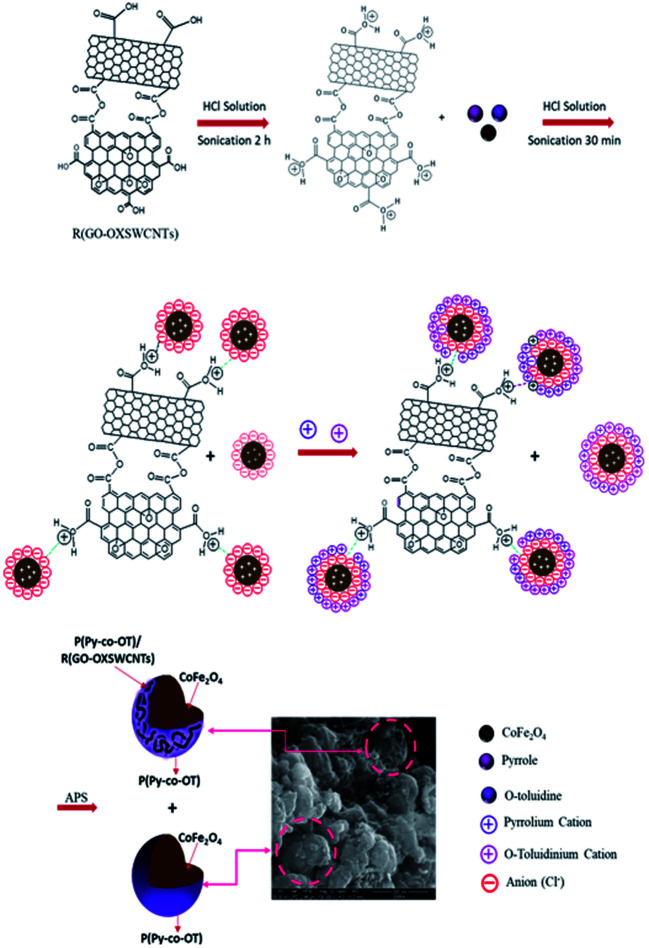
Illustration of the fabrication procedure of P(Py-*co*-OT)/CF/R(GO–OXSWCNTs) NCs.

First, 5% R(GO–OXSWCNTs) NCs were added to a three-neck flask (250 mL) containing 100 mL of 1 M HCl; the mixture was ultrasonicated for 2 h at room temperature. This was followed by addition of 5% of the CF NPs, 0.67 mL of doubly distilled Py and 1.07 mL of doubly distilled OT. The mixture was ultrasonicated for 30 min. Then, the mixed solution was cooled to 0–4 °C in an ice bath with continuous stirring. A pre-cooled solution of (5 g) ammonium persulfate (APS) dissolved in 100 mL of 1 M HCl was then added dropwise into the above solution within approximately 30 min with constant stirring at 0–4 °C under a nitrogen atmosphere. The polymerization reaction was maintained at 0–4 °C in the nitrogen atmosphere for 24 h with constant stirring. The precipitate formed was collected by ultracentrifugation and washed with deionized water several times until the filtrate became colourless. Finally, the fine black powder was dried at 60 °C for 24 h. The synthesis route of P(Py-*co*-OT)/CF/R(GO–OXSWCNTs) NCs is presented in Scheme 1.

### 2.6. Fabrication of the electrode with a GCE and ternary P(Py-*co*-OT)/CF/R(GO–OXSWCNTs) NCs

To effective the electrode fabrication process, a slurry of P(Py-*co*-OT)/CF/R(GO–OXSWCNTs) NCs in ethanol was made and used to deposit on GCE in the form of a thin uniform layer. Then, the recently modified GCE was kept at ambient conditions until dry. The stability of the modified GCE was enhanced by the addition of drop of Nafion, commercially available as 5% suspension in ethanol. After that, it was placed inside a low-temperature oven at 35 °C until the electrode is dried completely. It should be mentioned that Nafion is not only improved the binding strength but also increased the electron transfer rate as well as conductance of the electrode, which described in our previous reports.^70–73^ The proposed electrochemical cell was assembled using a Keithley electrometer. The working electrode was P(Py-*co*-OT)/CF/R(GO–OXSWCNTs) NCs/binder/GCE with a simple Pt wire as the counter electrode. The whole study was performed in a phosphate buffer medium with pH 7.0, which was formed by the mixing equimolar concentrations of monosodium and disodium phosphate buffer solutions. To examine the selectivity of the desired sensor, several toxic heavy metal ions were analysed in an electrochemical (*I*–*V*) approach in phosphate buffer medium very carefully. The stock solution of Ga^3+^ ions was diluted to form a range of Ga^3+^ ion solution concentrations of 0.1 mM to 0.1 nM. Then, the prepared Ga^3+^ ion solution was subjected as an analyte to execute the calibration curve of the proposed Ga^3+^ ion sensor. The sensor sensitivity was measured from the slope of the calibration curve and surface area of GCE (0.0316 cm^2^). The linear dynamic range (LDR) was defined as the range of the concentration of Ga^3+^ ion on the calibration curve considering the regression co-efficient (*R*^2^) value as a maximum. At a signal-to-noise ratio of 3, the detection limit of the proposed Ga^3+^ ion sensor was calculated. The examination was executed using phosphate buffer solution as a 10.0 mL constant during the electrochemical (*I*–*V*) investigation in an assembled electrochemical sensor. The thickness of fabricated film onto GCE is 0.5 μm.

### 2.7. Measurements and characterization

The morphologies and element distributions of the materials under study were observed by field emission scanning electron microscopy (SEM Model Quanta 250 FEG, with 30 kV accelerating voltage at 14× magnification up to 1 000 000 and resolution for Gun.1n) and high-resolution transmission electron microscopy (HR-TEM) (EM-2100, at 25× magnification and 200 kV) to provide fundamental structural and crystallographic information about the NCs. AFM, using instrument model 5600LS manufactured by Agilent Technology Company (USA), was used to observe the morphology of the NCs. X-ray diffraction (XRD) was also used to examine the NC crystallinity, carried out with a Bruker model D8 (including reflectometry, high-resolution diffraction, in-plane grazing incidence diffraction (IP-GID), small angle X-ray scattering (SAXS), as well as residual stress and texture investigations). Thermogravimetric analysis TGA, DTG and DTA tests were used to determine the thermal stability of the NC materials and to determine the degradation temperature; it was performed on a Shimadzu DTA-50 & TGA-50 system at a heating rate of 10 °C min^−1^ in an air atmosphere. To obtain functional groups information of the samples under study and to record the Fourier Transform Infrared (FTIR) spectra, a JASCO model was used within the range of 4000–300 cm^−1^. Raman measurements were carried out for samples using a Raman spectrometer (Lab. RAM-HR Evolution Horiba Co.) with a single visible spectrometer equipped with an air-cooled open electrode 1024 × 256 pixel CCD detector, a 532 nm He–Cd laser with 1800 grating (450–850 nm) and a 10% ND filter using an acquisition time of 5 s, 5 accumulations without spike filter and delay time, and a 100× objective. The electrical conductivities were measured by a four-probe resistivity instrument (SP4-62085TRY) using pressed pellets of sample powder with a thickness of approximately 0.2 mm and a diameter of 1 cm. A Keithley electrometer originated from USA works on two electrodes (working and counter electrodes) was used mainly as the investigation tool for this research.

### 2.8. Choice of materials

Here, P(Py-*co*-OT)/CF/R(GO–OXSWCNTs) NCs have employed a great deal of consideration due to their reticular branch structure, high strength to weight ratios, and functional properties in terms of large-active pores, large surface area, high-stability, high porosity, good conductivity (in terms of heat and electrical), economical processing, and permeability, which directly dependent on the structural and morphology properties. This nanocomposite preparation technique has several advantages including facile preparation, accurate control of the reactant temperature, easy to handle, and high-porosity as well as porous natures. Functional, optical, morphological, electrical, and chemical properties of P(Py-*co*-OT)/CF/R(GO–OXSWCNTs) nanocomposite are of huge significance from the scientific aspect, compared to other conventional composites. In this composites, SWCNT conjugated conducting polymer nanocomposites have also attracted considerable interest owing to their potential applications in fabricating sensors, electro-analytical devices, selective detection of bioassays, biological devices, hybrid-composites, electron-field emission sources for emission exhibits, biochemical detections, and surface-enhanced Raman properties *etc.* Therefore, a novel ternary conductive hybrid nanocomposite materials with high stability, high conductivity and excellent electrochemical properties has been developed in this approach. The composite material is based on CoFe_2_O_4_ (CF) as well as R(GO–OXSWCNTs) with reticular branch structures coated by the copolymer P(Py-*co*-OT) matrix. Here, the ternary P(Py-*co*-OT)/CF/R(GO–OXSWCNTs) NCs were synthesized *via* an *in situ* chemical polymerization method and eventually chosen this nanocomposite for selective and sensitive metal ionic sensor by electrochemical approach at room conditions.

## 3. Results and discussion

### 3.1. Nanocomposite fabrication

**Fig. 1:**
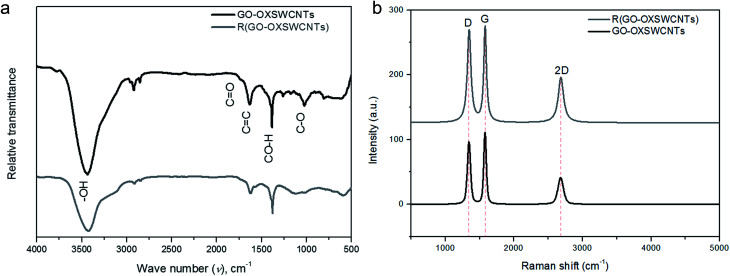
(a) FTIR spectra of GO–OXSWCNTs, R(GO–OXSWCNTs) NCs. (b) Raman spectra of GO–OXSWCNTs, R(GO–OXSWCNTs) NCs.

The inclusion of the R(GO–OXSWCNTs) with the reticular branch structures gives the properties for depositing the nanoscale layers of CPs, such as a relatively large area and high conductivity. The enhancement of the conductivity occurs due to both charge transfer and transport of ions throughout the composites. FTIR spectroscopy gives specific information about functional groups on a surface with the different molecular vibration modes. Fig. 1(a) displays the FTIR spectra of GO–OXSWCNTs and the R(GO–OXSWCNTs) NCs. In the GO–OXSWCNTs, broad peaks at 3440 cm^−1^ correspond to stretching vibrations of –OH.^65^ The peaks at 1632 cm^−1^ are due to the vibration of the unoxidized C=C skeletal structure, and those at 1383 cm^−1^ correspond to CO–H.^74^ The bands at 1732 cm^−1^ and 1025 cm^−1^ correspond to the C=O stretching vibration modes and C–O–C groups, respectively.^75^ Compared with the R(GO–OXSWCNTs), the R(GO–OXSWCNTs) sample shows the decreased intensity of both C=O as well as C–O bonds due to the reduction reaction, which confirmed the successful conversion of GO–OXSWCNTs during co-reduction processing. Raman spectra can exhibit differences in structure between the GO–OXSWCNTs and R(GO–OXSWCNTs). Fig. 1(b) displays the Raman spectra of GO–OXSWCNTs and R(GO–OXSWCNTs) NCs, which exhibit two dominant peaks near 1340 and 1576 cm^−1^ attributed to the D and G bands, respectively. The D band is ascribed to the breathing mode *k*-point phonons of *A*_1g_ symmetry with vibrations of the carbon atoms of the defected and disordered graphite. The G band is assigned to E_2g_ phonons of sp^2^ carbon atoms.^76–78^ The band at 2684 cm^−1^ occurs because of a D band overtone called the 2D band, which is present due to the presence of OXSWCNTs in NCs.^79^ The intensity ratio of the D/G bands for GO–OXSWCNTs was 0.87. After reduction, the *I*_D_/*I*_G_ for R(GO–OXSWCNTs) increased to 0.97 due to the restoration of sp^2^ carbons and decrease in the average sizes of sp^2^ domains upon reduction.^80,81^ Therefore, the enhanced value of *I*_D_/*I*_G_ of R(GO–OXSWCNTs) indicates the reduction of GO–OXSWCNTs.^82^ The higher intensity in the D band also suggests that a more isolated graphene domain was presented in R(GO–OXSWCNTs) compared to GO–OXSWCNTs and is also due to removal of oxygen moieties from GO–OXSWCNTs after reduction.^83,84^

### 3.2. Characterization & identification tools

**Fig. 2:**
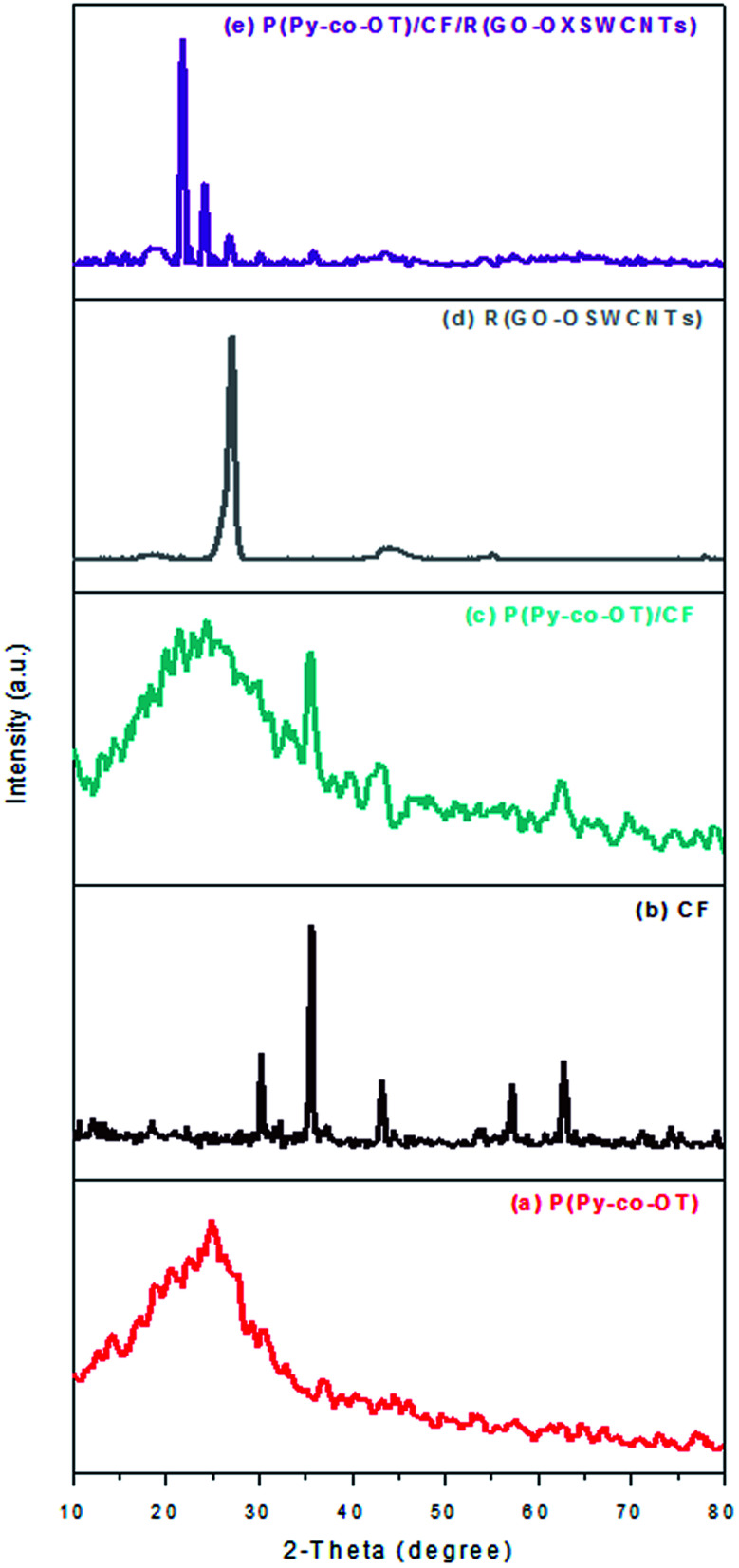
XRD patterns of P(Py-*co*-OT) (a), CF NPs (b), P(Py-*co*-OT)/CF NCs (c), R(GO–OXSWCNTs) (d) and P(Py-*co*-OT)/CF/R(GO–OXSWCNTs) NCs (e).

XRD was performed as a phase investigation of the crystallized products. Fig. 2 presents the diffraction patterns of XRD. The broad diffraction peak at the range 2*θ* = 15°–33° in Fig. 2(a) indicates the amorphous nature of the copolymer P(Py-*co*-OT) resulting from the interaction between the two different CPs.^85^ From the literature, the main diffraction peak at approximately 2*θ* = 25° and 20° represent periodicities perpendicular and parallel to the chains of the polymers, respectively.^86^ From Fig. 2(b), it can be seen that all the diffraction peaks of CF indicate the formation of single-phase CF in a cubic structure; no impurity peaks were observed (JCPDS 22-1086).^87^ The peaks at the 2*θ* values of 18.3°, 30.2°, 35.5°, 37.2°, 43.2°, 53.6°, 57.0° and 62.6° can be listed as the (111), (220), (311), (222) (400), (422), (511) and (440) CF crystal planes, respectively.^86,87^ The diffraction patterns of binary P(Py-*co*-OT)/CF NCs Fig. 2(c) contain the characteristic peaks of CF NPs as well as the broad diffraction peak of P(Py-*co*-OT). Furthermore, the CF peak intensity in these NCs is weaker than that of the pure CF, which indicates that the P(Py-*co*-OT) coating layer influences the peak intensity of CF NPs and causes the decrease in the ferrite crystallinity. These results indicate that P(Py-*co*-OT)/CF core–shell NCs are obtained. The R(GO–OXSWCNTs) NCs Fig. 2(d) showed peaks at approximately 2*θ* = 27.1° and 44.1°. The peak at approximately 2*θ* = 27.1° is produced by the introduction of CNTs and the partial conversion of GO to rGO.^65^ The XRD pattern of the ternary P(Py-*co*-OT)/CF/R(GO–OXSWCNTs) NCs Fig. 2(e) shows peaks characteristic of P(Py-*co*-OT), CF and R(GO–OXSWCNTs), which confirms the formation of the ternary NCs. The slight shift in the peaks of CF and R(GO–OXSWCNTs) may be attributed to the presence of R(GO–OXSWCNTs) NCs. It is clear that in the ternary NCs, the characteristic diffraction peaks of P(Py-*co*-OT) are weak in comparison to the diffraction peaks of pristine P(Py-*co*-OT), which can be produced by the interactions among CF, R(GO–OXSWCNTs) and P(Py-*co*-OT). The peak intensities of CF and R(GO–OXSWCNTs) in these ternary NCs are weaker than those of CF NPs and R(GO–OXSWCNTs), which reveals that the CF NPs and R(GO–OXSWCNTs) are both coated by P(Py-*co*-OT).

**Fig. 3:**
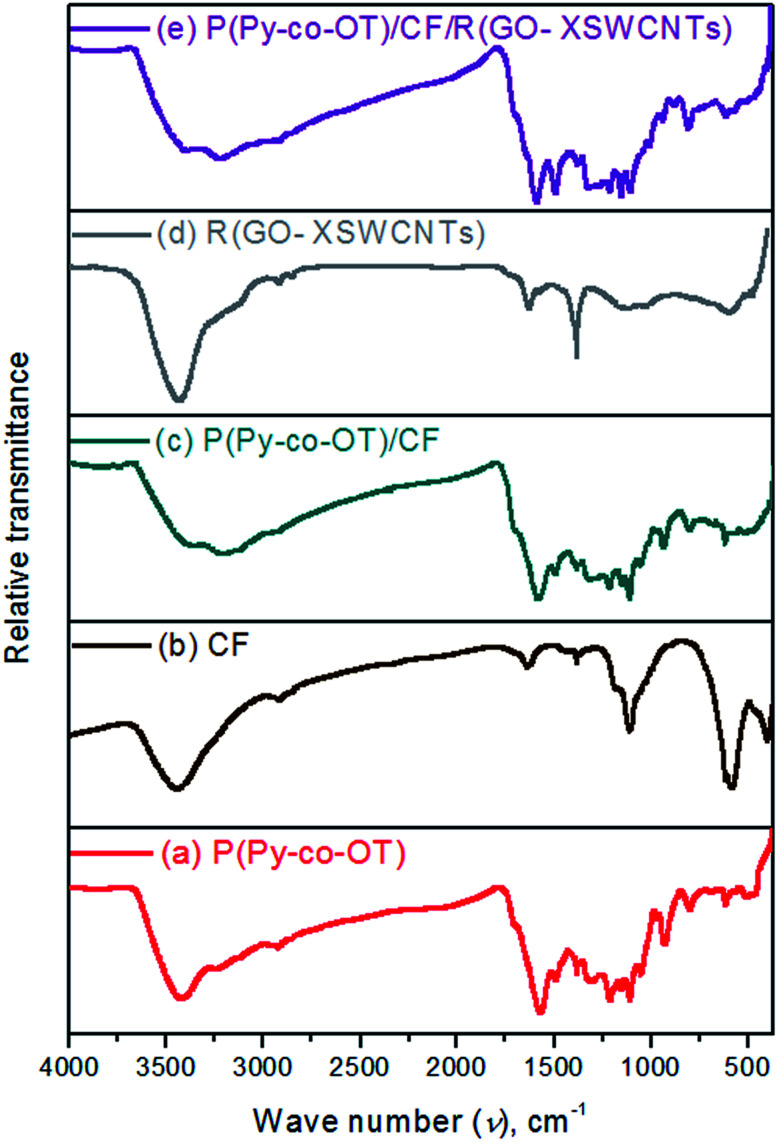
FTIR of P(Py-*co*-OT) (a), CF NPs (b), P(Py-*co*-OT)/CF NCs (c), R(GO–OXSWCNTs) (d) and P(Py-*co*-OT)/CF/R(GO–OXSWCNTs) NCs (e).

Fig. 3 shows the FTIR spectra of P(Py-*co*-OT), CF NPs, binary P(Py-*co*-OT)/CF NCs, R(GO–OXSWCNTs) and ternary P(Py-*co*-OT)/CF/R(GO–OXSWCNTs). From the literature, (–NH–) stretching vibrations of poly-*o*-toluidine POT appear at 3372 cm^−1^, and the (–NH–) vibration peak of polypyrrole PPy is hardly exhibited. From Fig. 3(a), a broad and weak band at 3526–3297 cm^−1^ is recognized as the characteristic (–NH–) stretching vibration, which indicates the appearance of a group of (–NH–) in Py as well as OT.^88,89^ The stretching vibrations of aromatic and aliphatic C–H appear at 3015, 2987, 2919 and 2852 cm^−1^. The bands at approximately 1569, 1212, 1049 and 928 cm^−1^ are assumed to be characteristics of Ppy. In Fig. 3(b), the two clear peaks at 585 cm^−1^ and 403 cm^−1^ are ascribed to the vibration of tetrahedral (Th) and octahedral (Oh) complexes, respectively, thus indicating the formation of the spinel ferrite structure of CF.^90^ The IR spectrum of curve Fig. 3(d) shows R(GO–OXSWCNTs) peaks. In the IR spectra of both binary Fig. 3(c) and ternary Fig. 3(d) NCs, the vibration peaks of CF show a redshift compared with the peaks of CF. In addition, the peaks of R(GO–OXSWCNTs) also show a redshift in ternary NCs compared with the peaks of R(GO–OXSWCNTs). Moreover, all the peaks analogous to P(Py-*co*-OT) essentially appear to confirm the successful formation of the binary and ternary NCs. The absorption peak's redshift of CF and R(GO–OXSWCNTs) in the NCs may be due to the coating effect of copolymer P(Py-*co*-OT). Therefore, the results of FTIR and XRD confirm the successful synthesis of the P(Py-*co*-OT)/CF/R(GO–OXSWCNTs) NCs with good crystallinity and some chemical interactions.

**Fig. 4:**
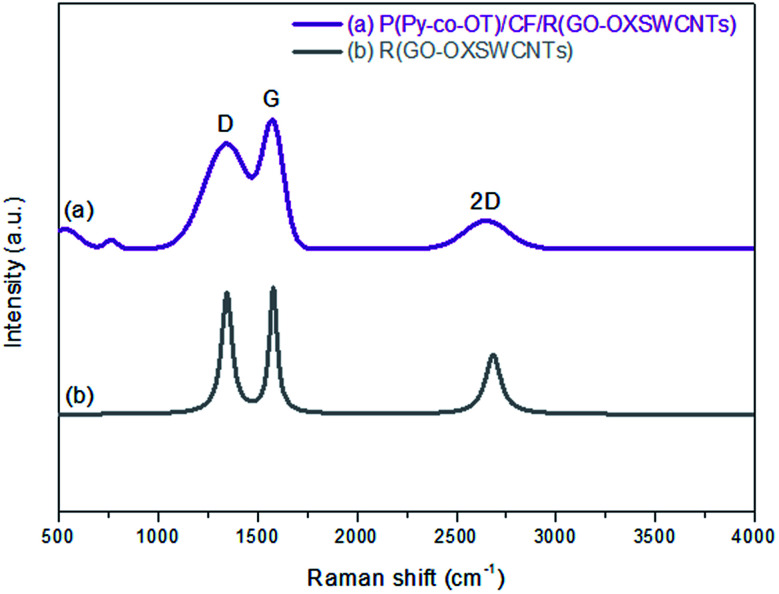
Raman spectra of R(GO–OXSWCNTs) (a) and P(Py-*co*-OT)/CF/R(GO–OXSWCNTs) NCs (b).

Raman spectroscopy is utilized to certify the presence of the R(GO–OXSWCNTs) side by side with CPs. Fig. 4 shows the Raman spectra of the P(Py-*co*-OT)/CF/R(GO–OXSWCNTs) NCs (a) and R(GO–OXSWCNTs) (b). The characteristic D and G peaks of R(GO–OXSWCNTs) appear in the Raman spectra of the P(Py-*co*-OT)/CF/R(GO–OXSWCNTs) NCs. Compared with the R(GO–OXSWCNTs) composite, a redshift in the G band can be observed, and there is no shift in the D band. The shifting might be due to a strong interaction between the polymer and R(GO–OXSWCNTs) and may indicate charge transfer among P(Py-*co*-OT), CF and R(GO–OXSWCNTs).^91^ However, the peaks centred at 534 and 764 cm^−1^ can be ascribed to the CF. These results confirm the presence of CF and R(GO–OXSWCNTs) in the as-synthesized composites.

**Fig. 5:**
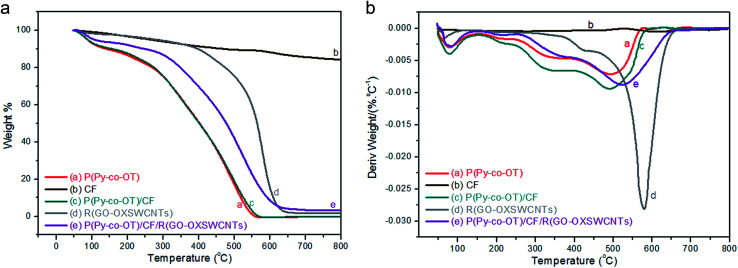
TGA (a) and DTG (b) curves of P(Py-*co*-OT) (a), CF NPs (b), P(Py-*co*-OT)/CF NCs (c), R(GO–OXSWCNTs) (d) and P(Py-*co*-OT)/CF/R(GO–OXSWCNTs) NCs (e).

**Fig. 6:**
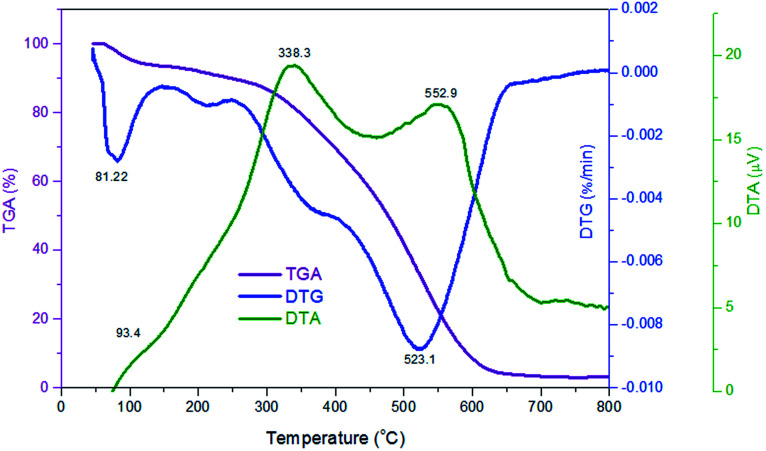
TGA, DTG and DTA curves of P(Py-*co*-OT)/CF/R(GO–OXSWCNTs) NCs.

TGA-DTG analyses of P(Py-*co*-OT) (a), CF NPs (b), binary P(Py-*co*-OT)/CF NCs (c), R(GO–OXSWCNTs) (d) and ternary P(Py-*co*-OT)/CF/R(GO–OXSWCNTs) NCs (e) are shown in Fig. 5(a). The thermogram of CF reveals a weight loss of 3%, which is due to solvent evaporation; 12% weight loss is noted at 576 °C due to decomposition of carbonaceous content present in the as-prepared CF.^92,93^ The stability of cobalt ferrite seems to be greater than that of the pure copolymer and other NCs. The TGA thermogram of P(Py-*co*-OT) shows a three-step weight loss. The initial mass loss of ∼10% at a lower temperature (less than 150 °C) is caused by the remaining water and HCl desorption. The second loss in mass of ∼25% is noted at 301 °C, possibly due to the volatilization of lower weight of P(Py-*co*-OT). The final loss in mass of ∼50% at higher temperatures (more than 402 °C) may be due to the thermal degradation of the P(Py-*co*-OT) chains. The binary P(Py-*co*-OT)/CF NCs are nearly identical to those of pure copolymer P(Py-*co*-OT). The ternary NCs shows three mass losses (∼10, 25 and 50% at 249, 371 and 477 °C, respectively), detailed in Table 1. This indicates that the stability of the ternary P(Py-*co*-OT)/CF/R(GO–OXSWCNTs) NCs is higher than that of the copolymer and binary NCs. The enhanced stability might result from the interactions among the P(Py-*co*-OT), CF and R(GO–OXSWCNTs). Additionally, CF NPs and R(GO–OXSWCNTs) are well coated by the P(Py-*co*-OT) chains. Further, from the DTG curves in Fig. 5(b), the CDT_max_ (the maximum composite degradation temperature)^94,95^ was determined and is listed in Table 1, which also indicates that the interaction between P(Py-*co*-OT), CF and R(GO–OXSWCNTs) increases the thermal stability of the ternary NCs. The final composite degradation temperature, CDT_final_, indicates the last temperature at which the decomposition finishes.^96,97^ The CDT_final_ values are determined from the TGA curve, as also shown in Table 1, showing that the CDT_final_ value of the P(Py-*co*-OT)/CF/R(GO–OXSWCNTs) NCs is the higher than copolymer P(Py-*co*-OT), binary P(Py-*co*-OT)/CF NCs, and R(GO–OXSWCNTs). In the DTA results in Fig. 6, ternary P(Py-*co*-OT)/CF/R(GO–OXSWCNTs) NCs have been observed to present two exothermic peaks at 338.3 °C (19 μV) and 552.9 °C (17 μV). The exothermic peak at 338.3 °C refers to the decomposition phase between 249–371 °C, whereas the exothermic peak at 552.9 °C refers to the second decomposition phase (371–477 °C).

**Table 1:** Thermal behaviour of P(Py-*co*-OT), binary P(Py-*co*-OT)/CF NCs, R(GO–OXSWCNTs) and ternary P(Py-*co*-OT)/CF/R(GO–OXSWCNTs) NCs

Sample	Temperature (°C) for various percentage decompositions^a^			CDT_max_^b^ (°C)	CDT_final_^a^ (°C)
	*T*_10_	*T*_25_	*T*_50_		
P(Py-*co*-OT)	150	301	402	489	565
P(Py-*co*-OT)/CF	150	301	402	490	578
P(Py-*co*-OT)/CF/R(GO–OXSWCNTs)	249	371	477	525	676
R(GO–OXSWCNTs)	395	501	563	579	662

a The values were determined by TGA at a heating rate of 10 °C min^−1^

b Determined from DTG curves.

**Fig. 7:**
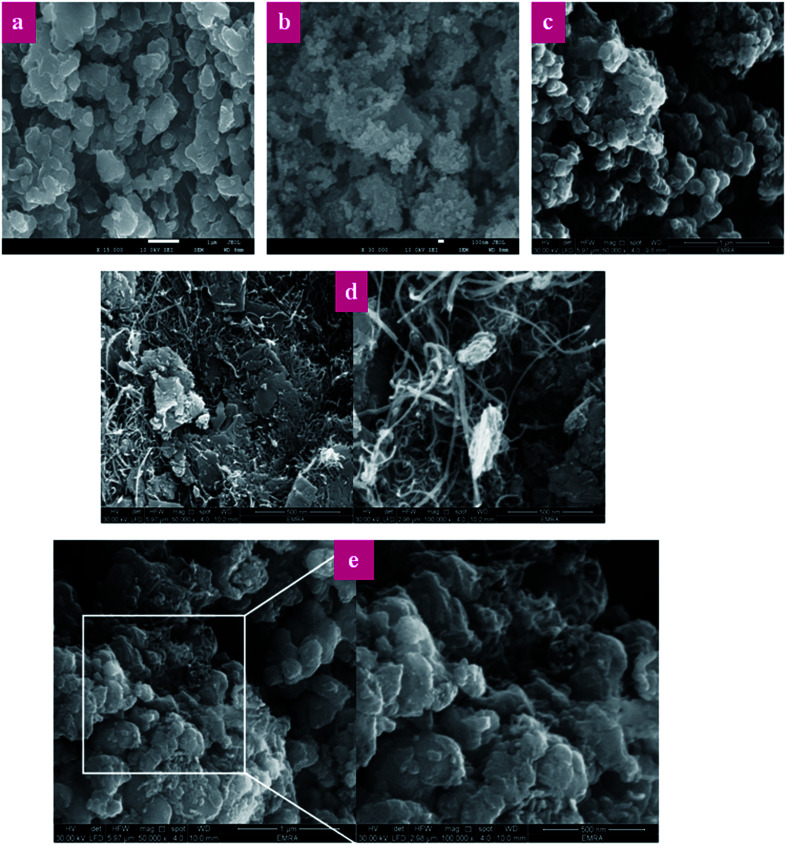
SEM images of P(Py-*co*-OT) (a), CF NPs (b), P(Py-*co*-OT)/CF NCs (c), R(GO–OXSWCNTs) (d) and P(Py-*co*-OT)/CF/R(GO–OXSWCNTs) NCs (e).

**Fig. 8:**
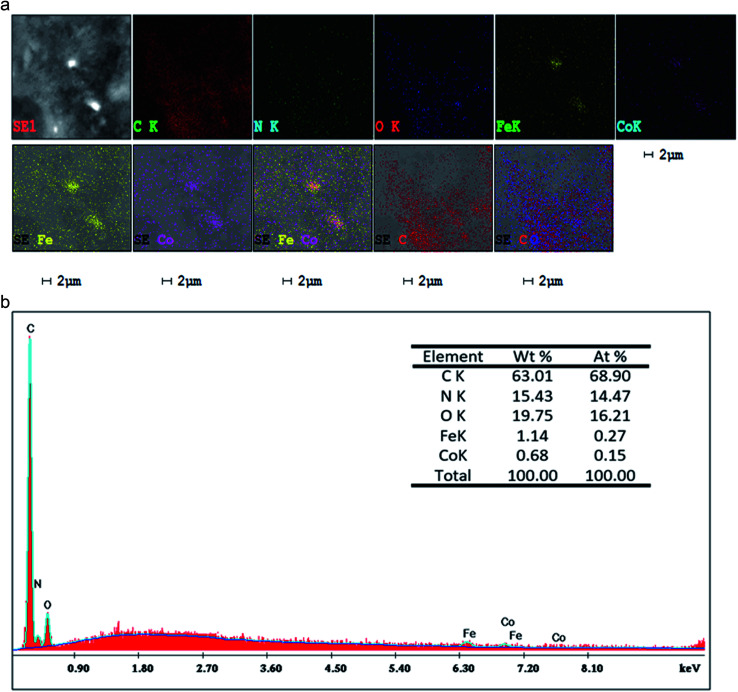
Corresponding elemental maps (a), EDX analyses of P(Py-*co*-OT)/CF/R(GO–OXSWCNTs) NCs (b).

**Fig. 9:**
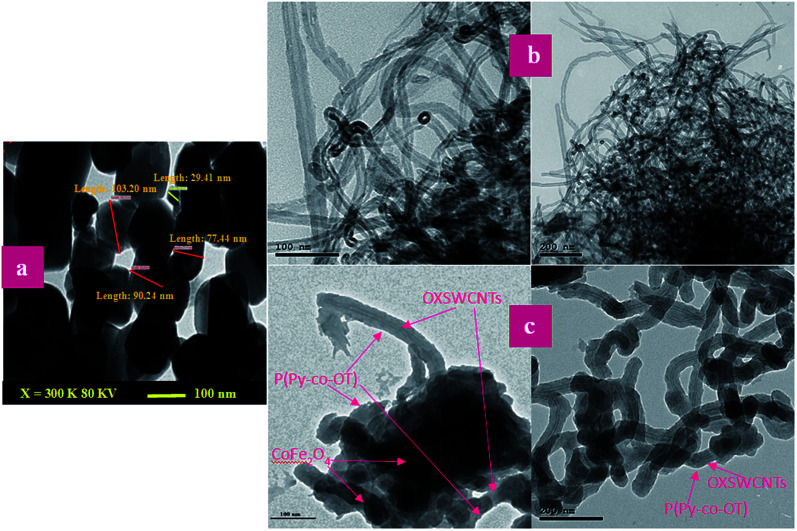
TEM images of CF (a), R(GO–OXSWCNTs) (b) and P(Py-*co*-OT)/CF/R(GO–OXSWCNTs) NCs (c).

**Fig. 10:**
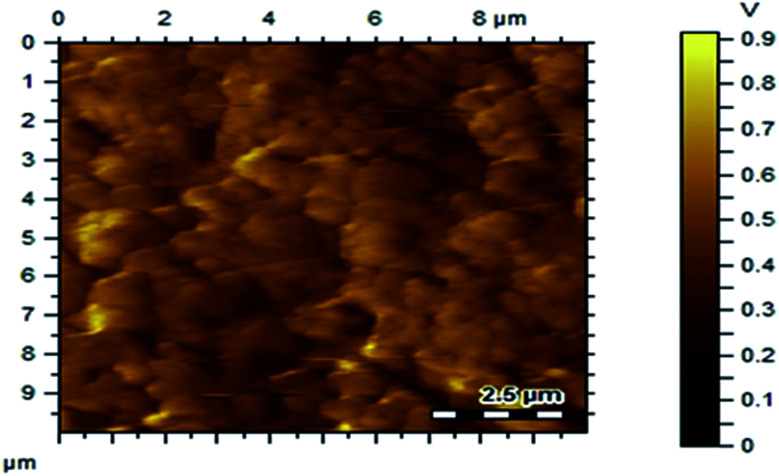
AFM images of P(Py-*co*-OT)/CF/R(GO–OXSWCNTs) NCs.

Fig. 7 shows the SEM images of P(Py-*co*-OT), pure CF NPs, binary P(Py-*co*-OT)/CF, R(GO–OXSWCNTs) and ternary P(Py-*co*-OT)/CF/R(GO–OXSWCNTs) NCs. Fig. 7(a) shows that the copolymer P(Py-*co*-OT) presents an irregular morphology. On the other hand, the CF NPs in Fig. 7(b) shows agglomerated nano-crystals having irregular shapes and sizes. Fig. 7(c) shows the SEM image of binary P(Py-*co*-OT)/CF. The morphology is similar to a globular agglomerate, indicating that the CF NPs are surrounded by polymer chains of the copolymer P(Py-*co*-OT). In other words, the polymer is coated on the surface of CF NPs because of the *in situ* polymerization. Fig. 7(d) shows the R(GO–OXSWCNTs) NCs; OXSWCNTs, which appear to be tubular, and the long nanotubes wrapped together, were uniformly dispersed on the plate-like sheets of RGO, the morphologies of which have irregular edges. The SEM image of the ternary P(Py-*co*-OT)/CF/R(GO–OXSWCNTs) NCs displayed in Fig. 7(e) shows that the P(Py-*co*-OT) polymerized and coated on the surface of CF and R(GO–OXSWCNTs), which appear as the globular agglomerate, with uniformly dispersed R(GO–OXSWCNTs) on the surface. These may be ascribed to the fact that R(GO–OXSWCNTs) NCs were dispersed on the surface of the CF nanosphere and then coated by a polymer layer. Fig. 8(a) shows the elemental mapping of the ternary P(Py-*co*-OT)/CF/R(GO–OXSWCNTs) NCs; the elements Co, Fe, C, O and N are uniformly distributed in the NCs, confirming the presence of CF and P(Py-*co*-OT) copolymer. As Fig. 8(b) shows, the EDX for the P(Py-*co*-OT)/CF/R(GO–OXSWCNTs) NCs shows the peaks corresponding to Co, Fe, C, O and N, confirming the presence of the CF and P(Py-*co*-OT) copolymer. Fig. 9(a) shows the TEM images of CF, which show nearly spherical agglomerated nanoparticles. This agglomeration phenomenon might be ascribed to the magnetic dipole interactions between particles with high surface energy. The R(GO–OXSWCNTs) are shown in Fig. 9(b); the tangled rope-like tubes with a smooth surface of OXSWCNTs can be observed to be uniformly distributed on the RGO sheets. Fig. 9(c) shows that the black core of aspherical CF NCs is coated by P(Py-*co*-OT). Because CF is the only crystalline material in the NCs, it can be concluded that CF NPs are present. At the same time, the diameter of ROXSWCNTs in P(Py-*co*-OT)/CF/R(GO–OXSWCNTs) NCs increased compared with those of R(GO–OXSWCNTs), signifying that a thin polymer layer was coated on the surface of R(GO–OXSWCNTs). No RGO was observed in these NCs. It can be concluded that RGO was completely covered by P(Py-*co*-OT). AFM images (Fig. 10) showed the morphology of the surfaces of the P(Py-*co*-OT)/CF/R(GO–OXSWCNTs) NCs to be a homogeneous exterior with spherical particles of regular size as well as uniform distribution. The CF NPs and R(GO–OXSWCNTs) are surrounded and embedded into the copolymer chain of P(Py-*co*-OT), which indicates that the CF NPs have a core influence on the polymerization of copolymer P(Py-*co*-OT).

**Fig. 11:**
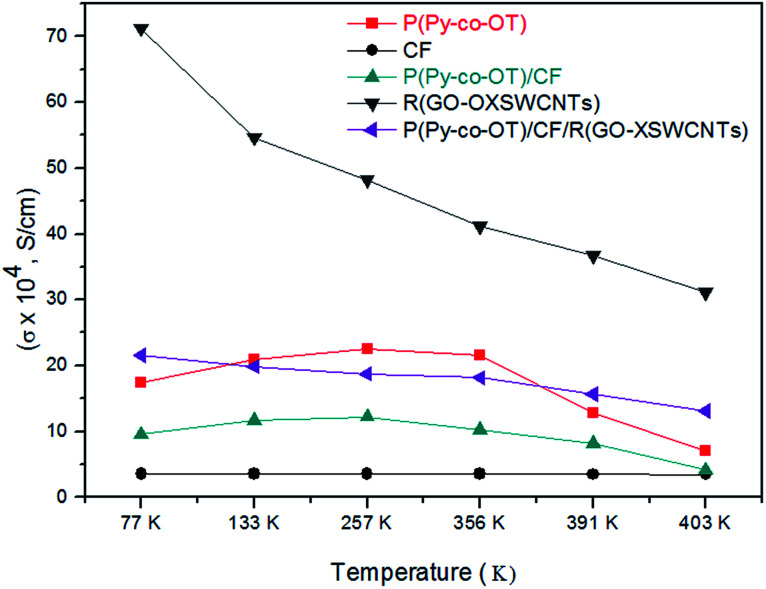
Temperature dependence of DC conductivity for P(Py-*co*-OT), CF NPs, P(Py-*co*-OT)/CF NCs, R(GO–OXSWCNTs) and P(Py-*co*-OT)/CF/R(GO–OXSWCNTs) NCs.

Table 2 shows the electrical conductivity of the samples. It is well known that PPy and POT are CPs and essentially conductive in nature because their structure has a conjugated π electron system. The carbon material is a class of conducting material, whereas the ferrite particles are insulators. From Fig. 11, it can be recognized that the conductivity of the R(GO–OXSWCNTs) is exhibited the highest values compared to other samples, while the conductivity of CF is the least of them. The conductivity of the NCs is strongly affected by the CF and R(GO–OXSWCNTs) contents. The introduction of CF NPs will impact the copolymerization of the conductive copolymers and further induce destruction of the regularity and continuity of the conjugated chains of copolymers. Furthermore, blockage of the carrier migration channel on the copolymer chains causes a decrease in conductivity. Additionally, some interaction and potential bonding effect between CF NPs and copolymer chains will decrease the electronic density of the copolymer chains and result in the decrease of the conductivity of the NCs. Additionally, any interaction and potential bonding effect between CF NPs and copolymer chains will cause a decrease the electronic density of the copolymer chains and result in a decrease of NCS conductivity, as observed in binary P(Py-*co*-OT)/CF NCs. In ternary P(Py-*co*-OT)/CF/R(GO–OXSWCNTs) NCs, the increase in conductivity can be ascribed to the highly conductive nature of the R(GO–OXSWCNTs). Furthermore, the enhanced conductivities of the NCs also benefit from the conducting bridge impact served by the special reticular branch structures of R(GO–OXSWCNTs) and charge transfer from P(Py-*co*-OT) to the R(GO–OXSWCNTs). In Fig. 11, the semiconducting behaviours of copolymer P(Py-*co*-OT) (a) and binary NCs (c) are measured very clearly. In the lower temperature range, the conductivity increases with increasing temperature, whereas the conductivity decreases little with increasing temperature at the higher temperature range, exhibiting metallic behaviour. The apparent decrease in conductivity at higher temperatures may be ascribed to the supremacy of the phonon-electron collision over the mobility impact. The conductivity of ternary NCs (e) presented a similar conducting behaviour (metallic behaviour) to that of R(GO–OXSWCNTs) (d), wherein, with increasing temperature, the conductivity decreases slightly. The noticeable decrease in conductivity at higher temperatures might be ascribed to starting the thermal dissociation of the samples, as confirmed by thermal analyses.

**Table 2:** Temperature dependence of DC conductivity for P(Py-*co*-OT), CF, P(Py-*co*-OT)/CF, R(GO–OXSWCNTs) and P(Py-*co*-OT)/CF/R(GO–OXSWCNTs) NCs

samples	Conductivity × 10^4^ (*σ*, S cm^−1^)					
	77 K	133 K	257 K	356 K	391 K	403 K
P(Py-*co*-OT)	17.38193322	20.90440832	22.57163107	21.55767108	12.84927543	7.043731713
CoFe_2_O_4_	3.611756412	3.558197161	3.543347542	3.535402817	3.519619768	3.466560174
P(Py-*co*-OT)/CF	9.548034251	11.67992338	12.2503081	10.33476366	8.200243383	4.113875361
R(GO–OXSWCNTs)	71.20985544	54.64122571	48.19881045	41.18480445	36.66952689	31.17945648
P(Py-*co*-OT)/CF/R(GO–OXSWCNTs)	21.55767108	19.88743711	18.77130543	18.15382828	15.67830624	13.06216283

### 3.3. Mechanism of action

Depending on the previous analysis, a proposed scheme for the formation of novel hybrid NCs is suggested to explain the preparation process of the ternary P(Py-*co*-OT)/CF/R(GO–OXSWCNTs) NCs (Scheme 1). As reported by the charge compensation mechanism, because of the polymerization in the acidic environment, the ferrite NPs surface gained a positive charge. In addition, the –COOH groups on the R(GO–OXSWCNTs) surface tend to be protonated, which means they gain H^+^ from the medium.^98^ Thus, the adsorption of a number of anions, for instance, Cl^−^, may compensate for the positive charges on the surfaces of ferrite and R(GO–OXSWCNTs). Furthermore, in this system of charge compensation, extra adsorption of the Cl^−^ on the ferrite and R(GO–OXSWCNTs) surface could work as the charge compensator for positively charged P(Py-*co*-OT) chains in the formation of P(Py-*co*-OT)/CF/R(GO–OXSWCNTs) NCs. As Py and OT monomers are shifted to pyrrolium cation and *o*-toluidinium cation ions, respectively, under acidic environments, electrostatic interactions take place between anions absorbed on the surface of ferrite and R(GO–OXSWCNTs) and pyrrolium cation and *o*-toluidinium cation ions. In addition, it is also possible that three types of hydrogen bonding will appear: first, between the oxygen atoms on the surface of ferrite and P(Py-*co*-OT) chains; second, hydrogen bonding occurs between the oxygen atoms on the surface of R(GO–OXSWCNTs) and P(Py-*co*-OT) chains; finally, the hydrogen bonding between the P(Py-*co*-OT) chains also happens in the core–shell NCs. Finally, the π–π stacking between the aromatic rings of P(Py-*co*-OT) and the π bonds of R(GO–OXSWCNTs) further stabilizes the bound complex structure of the composite. The interactions can confirm ferrite particles and R(GO–OXSWCNTs) to be inserted into the copolymer chains and produce core–shell structures^99–101^

### 3.4. Detection of Ga^3+^ by P(Py-*co*-OT)/CF/R(GO–OXSWCNTs) NCs/GCE

**Fig. 12:**
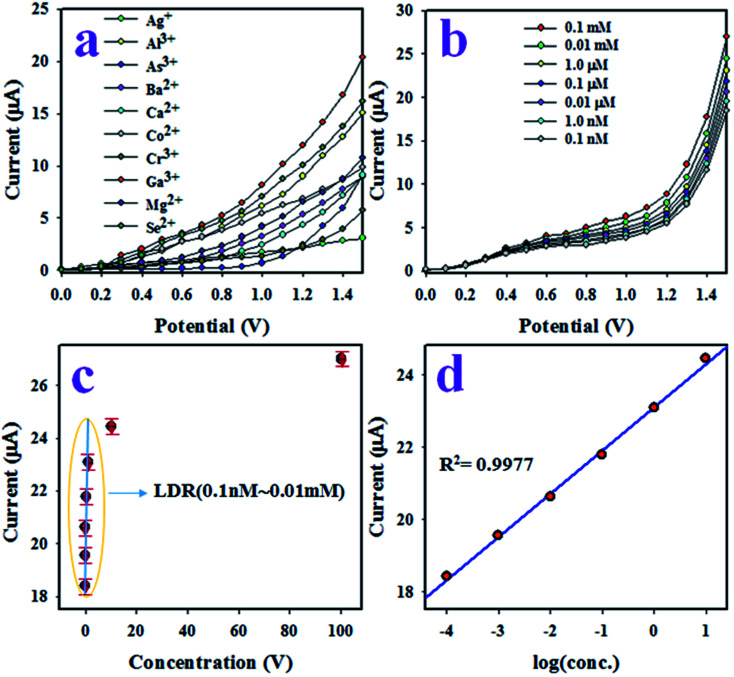
The electrochemical (*I*–*V*) characterization of sensor based on P(Py-*co*-OT)/CF/R(GO–OXSWCNTs) NCs/binder/GCE. (a) The identification of selectivity, (b) the variation of *I*–*V* responses of Ga^3+^ ion with a variation of concentration from lower to higher, (c) the calibration of Ga^3+^ ion sensor and (d) log(conc.) *vs.* current.

**Fig. 13:**
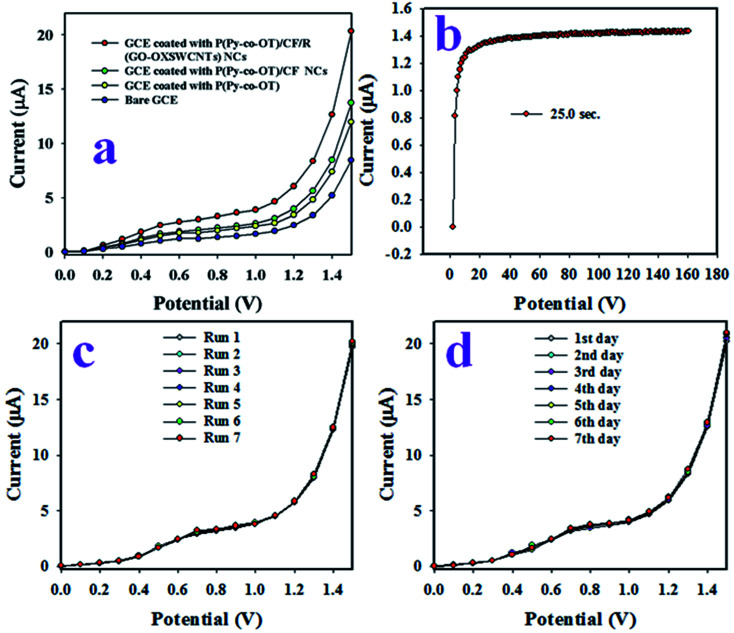
(a) The comparison study of Ga^3+^ ion sensor, (b) response time, (c) reproducibility and (d) stability test.

The aim of this study was the development of an electrochemical sensor with P(Py-*co*-OT)/CF/R(GO–OXSWCNTs) NCs deposited on a GCE for reliable and quick detection of hazardous and toxic heavy metal ions of Ga^3+^ in phosphate buffer medium (PBS) of pH 7.0. The assembled P(Py-*co*-OT)/CF/R(GO–OXSWCNTs) NCs/binder/GCE sensor has shown several useful benefits, including long-term stability in PBS, enhanced electrochemical (*I*–*V*) activities, inertness in a chemical environment, simple assembly and, above all, a safe chemical nature. Thus, the Ga^3+^ ion sensor based on P(Py-*co*-OT)/CF/R(GO–OXSWCNTs) NCs/binder/GCE was used successfully to quantify Ga^3+^ ions. The *I*–*V* was measured on the thin film of P(Py-*co*-OT)/CF/R(GO–OXSWCNTs) NCs/binder/GCE and the holding period in the electrometer was set as 1 s during the sensing performance of Ga^3+^ ion by assembling the electrochemical sensor. At the beginning of this study, a number of heavy and environmentally toxics ions were analysed by an assembled electrochemical sensor based on P(Py-*co*-OT)/CF/R(GO–OXSWCNTs) NCs/binder/GCE at an applied potential 0 to +1.5 V and concentration level of each metal ion of 0.1 μM. Fig. 12(a) shows the electrochemical (*I*–*V*) responses of Ag^+^, Al^3+^, As^3+^, Ba^2+^, Ca^2+^, Co^2+^, Cr^3+^, Ga^3+^, Mg^2+^ and Se^2+^; among these, Ga^3+^ ion shows the highest *I*–*V* response. Therefore, based on highest *I*–*V* response, the Ga^3+^ cation was considered as selective to the P(Py-*co*-OT)/CF/R(GO–OXSWCNTs) NCs/binder/GCE sensor. To inspect the electrochemical behaviour of the Ga^3+^ ion sensor in terms of linear dynamic range, sensitivity and detection limit, the proposed sensor was used to analyse Ga^3+^ ions based on concentrations from lower to higher, as illustrated in Fig. 12(b). Fig. 12(b) shows that *I*–*V* responses increased with increasing of concentration of Ga^3+^ ions; similar observations have been reported by previous authors for the detection of environmental toxic chemicals in PBS.^102–106^ The calibration of the Ga^3+^ cation sensor was performed, as shown in Fig. 12(c). To execute this calibration, the current data from Fig. 12(b) are isolated at an applied potential +1.5 V, and a new plot, Fig. 12(c), is plotted as current *vs.* concentration of Ga^3+^ ions. As Fig. 12(c) shows, the current data are continuously distributed from 0.1 nM to 0.01 mM in a linear manner, and this linear plot is fitted with the regression coefficient of *R*^2^ = 0.9977, as shown in Fig. 12(d) current *vs.* log(conc.) plot. Therefore, the concentration range of 0.1 nM to 0.01 mM is defined as the linear dynamic range (LDR) of Ga^3+^ ions by proposed Ga^3+^ ion sensors based on the P(Py-*co*-OT)/CF/R(GO–OXSWCNTs) NCs/binder/GCE. The sensitivity of the Ga^3+^ cationic sensor is calculated from the slope of the calibration curve in the LDR range, and it is found to be 13.0569 μA μM^−1^ cm^−2^, a potentially efficient and appreciable sensitivity of the Ga^3+^ ion sensor. Applying a signal-to-noise ratio of 3, the detection limit (DL) of the cationic sensor is estimated as 96.27 ± 4.81 pM, definitely a lower limit of detection. Quantification limit (43.523 pM) is also calculated. Fig. 13(a) shows the electrochemical responses of the GCE modified with various sensing elements, such as pure copolymer P(Py-*co*-OT), P(Py-*co*-OT)/CF, and P(Py-*co*-OT)/CF/R(GO–OXSWCNTs) NCs. As Fig. 13(a) shows, the modified GCE with P(Py-*co*-OT)/CF/R(GO–OXSWCNTs) NCs exhibits the maximum *I*–*V* response, whereas the bare GCE is the lowest. The experiment was conducted at an applied potential of 0 to +1.5 V and 0.1 μM of Ga^3+^ ion in PBS. Therefore, it is confirmed that the sensing element, the P(Py-*co*-OT)/CF/R(GO–OXSWCNTs) NCs, has the maximum affinity to Ga^3+^ ions. The response time is an important analytical property of an electrochemical sensor, and it is measured in time (seconds). The proposed Ga^3+^ cationic sensor was used to investigate the response time, as demonstrated in Fig. 13(b). This performance was executed with 0.1 μM Ga^3+^ ion in PBS. The resulting response time was found to be approximately 25.0 s. Moreover, this amount of time is required by the projected Ga^3+^ ion sensor based on P(Py-*co*-OT)/CF/R(GO–OXSWCNTs) NCs/binder/GCE to complete the cycle of analysis and, obviously, the obtained response time is very short and appreciable. The measurement of Ga^3+^ ions in an identical conditions with a similar outcome is defined by the reproducibility or reproducibility performance of the electrochemical fabricated sensor. This test was carried out with 0.1 μM Ga^3+^ ions and potential ranging as 0 to +1.5 V in PBS, presented in Fig. 13(c). As in Fig. 13(c), the seven runs are completely replicated. The indistinguishable results are obtained. To identify the precision of this reproducibility test, the current data at potential +1.5 V from Fig. 13(c) were used to measure the percentage of relative standard deviation (%RSD). It is equal to 0.84%, providing evidence of high precision. Therefore, it can be predicted that the proposed Ga^3+^ cationic sensor is reusable and reliable in real applications. To check the performance stability of the proposed Ga^3+^ ion sensor, a similar reproducibility test as in Fig. 13(c) was executed for seven days under similar conditions. The finding of this stability test is similar to the reproducibility test. Thus, it can be concluded that the proposed Ga^3+^ ion sensor has long-term stability to perform successfully in PBS. As demonstrated in Fig. 12(b), the electrochemical (*I*–*V*) performances are varied directly with the corresponding concentration of Ga^3+^ ions. In analysing the test of Ga^3+^ ions, the current data are measured on the thin film of P(Py-*co*-OT)/CF/R(GO–OXSWCNTs) NCs/binder/GCE. In the initial few seconds of the detection method of Ga^3+^ ions, where the Ga^3+^ ion is absorbed on the surface of a thin film of P(Py-*co*-OT)/CF/R(GO–OXSWCNTs) NCs/binder/GCE, the corresponding surface coverage is very small, and a reaction occurs. Due to enrichment of Ga^3+^ ions in PBS, the rate of reaction and surface coverage are enhanced and approach steady-state saturation. With the further increase of Ga^3+^ ion concentration, a steady-state equilibrium *I*–*V* response is perceived in Fig. 12(c), as the current data are continuously distributed in a linear manner. Thus, this observation provides information about the reliability of the method to detect real environmental samples successfully. The response time of the proposed Ga^3+^ cation sensor is 25.0 s. This means that the sensor requires 25.0 s to complete the detection of Ga^3+^ ions in PBS. To determine the value of this research, a comparison between the similar studies is necessary. Therefore, a comparison between the analytical properties, such as sensitivity, DL and LDR, is presented in Table 1.^25,107–114^ As stated in Table 3, the performances in this research are quite appreciable.

**Table 3:** Comparison of P(Py-*co*-OT)/CF/R(GO–OXSWCNTs)/Nafion/GCE sensor performance with various modified materials by different methods for the detection of Ga^3+^ cation^a^

Materials/methods	DL	LDR	Sensitivity	Ref.
MBBSH/GCE (*I*–*V*)	84.0 pM	0.1 nM to 1.0 mM	0.949 μA μM^−1^ cm^−2^	25
PBDMBS/GCE (*I*–*V*)	20.0 pM	1.0 nM to 0.01 M	0.396 × 10^−2^ μA μM^−1^ cm^−2^	107
Naphthalimide (fluorescence)	—	—	—	108
Nitrogen–graphitic carbon dot (fluorescence)	—	0–20 μM	—	109
Arene-based fluorophores (fluorescence)	—	—	—	110
Polarography	0.01 μg mL^−1^	—	—	111
CIGS materials (electrochemical)	—	—	—	112
DDTCT (electrochemical)	—	1.45 × 10^−6^ to 0.1 mol L^−1^	—	113
Mercury electrode (stripping voltammetry)	4 ng Ga per L	—	—	114
P(Py-*co*-OT)/CF/R(GO–OXSWCNTs) NCs/GCE (*I*–*V*)	96.27 pM	0.1 nM to 0.01 mM	13.057 μA μM^−1^ cm^−2^	This work

a DL (detection limit), LDR (linear dynamic range), pM (picomole), mM (millimole).

### 3.5. Real sample analysis

The validation of fabricated P(Py-*co*-OT)/CF/R(GO–OXSWCNTs) NCs sensor is applied to detect the target Ga^3+^ ions in various real environmental samples by standard addition method. Therefore, the developed electrochemical sensor was applied in the recovery method to test with various real environmental samples as well as industrial water, mineral water, sea water and tap water. The real samples are collected from the Jeddah city located in Saudi Arabia. Collected real samples are properly filtered and prepared for the analysis in laboratory. The results data are presented in Table 4, and the sensor is found to be quite satisfactory.

**Table 4:** The analysis of real environmental samples using P(Py-*co*-OT)/CF/R(GO–OXSWCNTs) NCs/GCE chemical sensor by recovery method

Sample	Added Ga^3+^ ion concentration (μM)	Measured Ga^3+^ ion conc.^a^ by P(Py-*co*-OT)/CF/R(GO–OXSWCNTs) NCs/binder/GCE (μM)			Average recovery^b^ (%)	RSD^c^ (%) (*n* = 3)
		*R*_1_	*R*_2_	*R*_3_		
Well water	0.01	0.00992	0.00998	0.00998	99.60	0.35
Mineral water	0.01	0.00993	0.00997	0.01001	99.67	0.40
Tap water	0.01	0.00990	0.00981	0.00985	98.53	0.46

a Mean of three repeated determination (signal to noise ratio 3) P(Py-*co*-OT)/CF/R(GO–OXSWCNTs) NCs/binder/GCE.

b Concentration of Ga^3+^ determined/concentration taken (unit: nM).

c Relative standard deviation value indicates precision among three repeated measurements (*R*_1_, *R*_2_ and *R*_3_).

## 4. Conclusion

In this study, we synthesized P(Py-*co*-OT)/CF/R(GO–OXSWCNTs) NCs as ternary NCs for sensor applications *via in situ* polymerization. First, FTIR and Raman analysis demonstrate the formation of the R(GO–OXSWCNTs). The addition of R(GO–OXSWCNTs) as the conducting frameworks for sustaining CF and P(Py-*co*-OT) may not only improve the electrical conductivity of the ternary NCs but also increase the thermostability and enhance the electrochemical properties of ternary NCs. The XRD, FTIR and Raman analyses demonstrate the formation of ternary NCs. Similarly, the SEM, TEM and AFM results revealed that the P(Py-*co*-OT) cover the CF NPs and R(GO–OXSWCNTs). The synthesized NCs of P(Py-*co*-OT)/CF/R(GO–OXSWCNTs) were applied as thin film on a GCE with a conductive Nafion binder to result in the working electrode of a gallium ion electrochemical sensor, which shows appreciable analytical characteristics, including sensitivity (13.0569 μA μM^−1^ cm^−2^), LDR (0.1 to 0.0 mM), DL (96.27 ± 4.81 pM), quantification limit (43.523 pM), response time (25.0 s), reproducibility and stability. In addition, the Ga^3+^ ion sensor proved efficient and reliable to analyse real environmental samples. This work indicates that P(Py-*co*-OT)/CF/R(GO–OXSWCNTs) NCs could be a promising electrochemical material for sensor applications.

## Conflicts of interest

There are no conflicts to declare.

## Supplementary Material
